# Supplementary material for: Shock Index Predicts Outcome in Patients with Suspected Sepsis or Community-Acquired Pneumonia: A Systematic Review
Source: J Clin Med. 2019 Jul 31;8(8):1144. doi: 10.3390/jcm8081144 (PMC6723191; doi:10.3390/jcm8081144)
Supplement: Supplementary file 1 [file jcm-08-01144-s001.pdf]

Shock index predicts outcome in patients with suspected sepsis or community acquired pneumonia: a systematic review

David J Middleton, Toby O Smith, Rachel Bedford, Mark Neilly, Phyo K Myint

Online Data Supplement

**Medline Search Strategy Example**
1. exp Emergency Treatment/<br>2. exp Emergency Medical Services/<br>3. exp Emergency Service, Hospital/<br>4. exp Crisis Intervention/<br>5. (crisis intervention or crisis interventions or critical incident or critical incidents).ab,ti.<br>6. (emergency medical service or emergency service*).ab,ti.<br>7. (emergicenter* or emergency care or emergency health services or emergency health service or emergency hospital service* or emergency unit or emergency room or emergency department or emergency health care or emergency medical care).ab,ti.<br>8. (emergency ward or emergency treatment or emergency therapy).ab,ti.<br>9. emergency medicine.ab,ti.<br>10. (acute care or acute medical care).ab,ti.<br>11. accident service.ab,ti.<br>12. (prehospital or pre-hospital or preclinical or pre-clinical adj3 care or support or treat*).ab,ti.<br>13. ambulance care.ab,ti.<br>14. exp First Aid/<br>15. exp Emergency Medical Technicians/<br>16. paramedic*.ab,ti.<br>17. ((emergency or critical or trauma or triage or ambulanc*) adj3 (doctor* or crew* or staff or team*)).ab,ti.<br>18. (Trauma Center* or Trauma Unit* or Triage*).ab,ti.<br>19. OR/1-18<br>20. exp Sepsis/<br>21. exp Shock,Septic/<br>22. (sepsis or septic shock).ab,ti.<br>23. (septic* or septicemia).ab,ti.<br>24. sepsis adj5 (urosepsis or severe pneumonia or gram negative or intra-abdominal)<br>25. OR/20-24<br>26. exp Mortality/<br>27. (Mortalit* or fatality rate* or death or survival).ab,ti.<br>28. (decreased function or functional status or functional impairment).ab,ti.<br>29. (adverse outcome* or poor outcome* or adverse health outcome*).ab,ti.<br>30. (readmission* or rehospitalization* or long term hospitalization or delayed discharge).ab,ti.<br>31. admission AND (critical care or critical care or intensive care or high dependency).ab,ti.<br>32. OR/26-31<br>33. Prognos*.ab,ti.<br>34. Predict*.ab,ti.<br>35. Incidence.sh.<br>36. Early recognition.ab,ti.<br>37. Utility.ab,ti.<br>38. Association.ab,ti.<br>39. Relationship.ab,ti.<br>40. OR/33-39<br>41. AND/19,25,32,40<br>42. (Shock Index).ab,ti.<br>43. AND/41,42

Table S1: Medline search strategy. The search strategy was modified as appropriate for each database.

	Baez 1990	Berger 2013	Chung 2018	Jaimes 2005	Jayarakash 2018	Lombaard 2015	Talmor 2007	Wira 2014	Yussof 2012	Curtain 2013	Eldaboosy 2015	Musonda 2011	Myint 2010	Nullman 2014	Sankaran 2011
1. Hypothesis/aims/objectives clearly stated	✓	✓	✓	✓	✓	✓	✓	✓	✓	✓	✓	✓	✓	✓	✓
2. Main outcome measures clearly stated	✓	✓	✓	✓	✓	✓	✓	x	✓	✓	✓	✓	✓	✓	✓
3. Characteristics of patients clearly described	x	✓	✓	✓	✓	✓	✓	✓	✓	✓	x	✓	✓	✓	✓
4. Interventions of interest clearly described	✓	✓	✓	✓	✓	✓	✓	✓	✓	✓	✓	✓	✓	✓	✓
5. Distribution of principle confounders clearly described	x	x	✓	✓	✓	x	✓	✓	x	x	x	x	x	x	x
6. Main findings clearly described	x	✓	✓	x	x	✓	x	✓	x	✓	x	✓	✓	✓	✓
7. Estimates of random variability in the data provided	✓	x	✓	✓	✓	✓	✓	✓	✓	✓	✓	✓	✓	✓	✓
8. Important adverse events reported	NA	NA	NA	NA	NA	NA	NA	NA	NA	NA	NA	NA	NA	NA	NA
9. Characteristics of patients lost to follow up described	✓	✓	x	x	x	✓	✓	✓	✓	✓	✓	✓	✓	✓	✓
10. Actual probability values reported	✓	✓	✓	✓	✓	✓	✓	✓	✓	✓	✓	✓	✓	x	✓
11. Participants approached representative of entire population	x	x	x	✓	✓	x	✓	x	x	✓	x	✓	✓	✓	✓
12. Participants recruited representative of the entire population	x	✓	✓	x	x	✓	✓	✓	✓	✓	x	✓	✓	✓	✓
13. Staff, places and facilities treated representative of the majority of population	x	x	x	x	x	x	x	x	x	x	x	✓	✓	x	✓
14. Blinding of study subjects	NA	NA	NA	NA	NA	NA	NA	NA	NA	NA	NA	NA	NA	NA	NA
15. Blinding of assessors	x	✓	x	x	✓	x	x	x	x	x	x	x	x	x	x
16. Data based on dredging clearly stated	✓	✓	✓	✓	✓	x	✓	x	✓	✓	x	✓	x	✓	✓
17. Time period between the intervention and outcome the same for cases and controls	✓	✓	✓	✓	✓	✓	✓	✓	x	✓	x	✓	✓	✓	✓
18. Appropriate statistical tests used	✓	✓	✓	✓	✓	x	✓	✓	✓	✓	x	✓	✓	✓	✓
19. Compliance to intervention reliable	NA	NA	NA	NA	NA	NA	NA	NA	NA	NA	NA	NA	NA	NA	NA
20. Main outcome measure reliable and valid	✓	✓	✓	✓	✓	✓	✓	✓	✓	✓	✓	✓	✓	✓	✓
21. Intervention groups or case-controls recruited from the same population	x	✓	✓	✓	✓	✓	✓	✓	✓	✓	✓	✓	✓	✓	✓
22. Intervention groups or case-controls recruited from the same time	x	✓	✓	✓	✓	✓	✓	✓	✓	✓	✓	✓	✓	✓	✓
23. Study subjects randomised to the interventions	NA	NA	NA	NA	NA	NA	NA	NA	NA	NA	NA	NA	NA	NA	NA
24. Was concealed randomisation to allocation undertaken	NA	NA	NA	NA	NA	NA	NA	NA	NA	NA	NA	NA	NA	NA	NA
25. Adequate adjustment of allocation made in analysis of confounders	x	x	x	✓	✓	x	✓	✓	x	x	x	x	x	x	x
26. Patient losses accounted for	✓	✓	x	✓	✓	✓	✓	✓	✓	✓	✓	✓	✓	✓	✓
27. Sufficiently powered cohort size	x	✓	x	✓	✓	x	✓	✓	x	x	x	✓	✓	✓	✓

Table S2: Downs and Black quality assessment for included studies.

Author / year	*n*	SI Threshold	Mortality (%)	Test characteristics for prediction of mortality				
				Sensitivity	Specificity	PPV	NPV	OR
Curtain et al 2013 (19)	95	≥ 1.0	8.4	0.88 (0.48-0.99)	0.72 (0.62-0.81)	0.23 (0.10-0.41)	0.98 (0.90-1.0)	18.3 (2.1-157.3)
Myint et al 2010 (30)	190	≥ 1.0	28.4	0.62 (0.47-0.75)	0.69 (0.56-0.79)	0.54 (0.41-0.68)	0.76 (0.65-0.85)	4.0 (2.1-7.9)
Nullmann et al 2014 (21)	553	≥ 1.0	10.7	0.42 (0.30-0.56)	0.81 (0.77-0.85)	0.21 (0.16-0.28)	0.92 (0.90-0.94)	3.2 (1.8-5.6)

Table S3: CURASI score as a predictor of mortality in CAP. A threshold value ≥ 2 was used for all studies.
